# Atopic Dermatitis and Atopic Keratoconjunctivitis: New Insights in the Analyses of Microbiota and Probiotic Effect

**DOI:** 10.3390/ijms26041463

**Published:** 2025-02-10

**Authors:** Francesco Petrillo, Annalisa Buonanno, Ludovica Fedi, Marilena Galdiero, Michele Reibaldi, Bruno Tamburini, Emilia Galdiero

**Affiliations:** 1 Department of Medical Sciences, Eye Clinic, Turin University, 10024 Turin, Italy; mreibaldi@libero.it; 2 Department of Biology, University of Naples “Federico II”, 80126 Naples, Italy; annalisa.buonanno@unina.it (A.B.); egaldier@unina.it (E.G.); 3 Department of Translational Medical Science, Section of Pediatrics, Università Degli Studi di Napoli Federico II, 80131 Naples, Italy; ludovicafedi1@gmail.com; 4 Department of Experimental Medicine, University of Campania “Luigi Vanvitelli”, 81100 Naples, Italy; marilena.galdiero@unicampania.it; 5 Department of Experimental Medicine, Università del Piemonte Orientale, 28100 Novara, Italy; 20042447@studenti.uniupo.it

**Keywords:** atopic dermatitis, keratoconjunctivitis, eczema, probiotics, gut microbiota

## Abstract

Atopy is defined as a predisposition to hypersensitivity reactions against a range of antigens. It is characterized by the activation of CD4+ T helper type 2 (Th2) cells and an increased production of immunoglobulin E (IgE). The most common atopic conditions are atopic dermatitis, asthma, allergic rhinitis, food allergies, and atopic ocular diseases. Atopic keratoconjunctivitis (AKC) is a chronic, bilateral inflammatory condition affecting the ocular surface, frequently occurring in conjunction with atopic dermatitis. It is not uncommon for patients to present with multiple conditions simultaneously or in a sequential manner. A comprehensive understanding of the underlying mechanisms of atopic diseases is essential for the effective clinical evaluation and treatment. Recent research has underscored the pivotal role of the microbiota in the pathogenesis of atopic dermatitis and atopic eye diseases, with alterations in microbial composition (dysbiosis) being linked to a spectrum of atopic conditions. Probiotics are currently being investigated as a potential treatment option for restoring microbial balance and alleviating disease symptoms. This review examines the relationship between atopic dermatitis, atopic keratoconjunctivitis, and the microbiota, evaluating the current evidence and exploring the potential of probiotics as a novel therapeutic approach.

## 1. Introduction

The term atopy is used to describe a tendency towards heightened immune responses to a variety of antigens or allergens. This leads to the differentiation of CD4+ T helper type 2 (Th2) cells and the excessive production of immunoglobulin E (IgE). These processes are responsible for the development of hypersensitivity reactions to a diverse range of allergens [1]. Atopic conditions are prevalent and include atopic dermatitis (eczema), asthma, allergic rhinitis, food allergies, and atopic eye diseases. It is possible for an individual to experience two or more of these conditions concurrently or at different times [1]. Understanding the physiopathological processes underlying atopic diseases is essential for accurate clinical assessment and effective treatment, thereby improving the care of those affected.

Recently, there has been increasing interest in the role of the microbiota in the development of allergic diseases. The human microbiota consists of a diverse array of microorganisms that fulfil protective and regulatory roles, contributing to immune balance and overall health [2]. Changes in the composition of these microbial communities, known as dysbiosis, have been associated with several atopic diseases [3]. In addition, the use of probiotics has been investigated as a potential therapeutic approach to restore the balance of the microbiota and may help alleviate the symptoms of the diseases, offering a promising strategy for the management of these conditions [4].

This review will focus specifically on atopic dermatitis and atopic keratoconjunctivitis due to their high prevalence and significant impact on patients’ quality of life. Indeed, Atopic dermatitis (AD)affects more than 25% of children and 10% of adults in the western world, while atopic eye diseases are known AD-associated comorbidities with combined incidence rates potentially reaching more than 55%. We also explore the links between atopic dermatitis, atopic keratoconjunctivitis and the microbiota, reviewing the current evidence and discussing the potential role of probiotics as a novel therapeutic option.

## 2. Atopic Dermatitis

AD, also known as atopic eczema, is a chronic inflammatory dermatological condition with a 10% lifetime prevalence. It is characterized by pruritic, erythematous, and eczematous lesions and affects a significant proportion of the world’s population, affecting up to 12% of children and 7.2% of adults, and contributes to significant health care utilization. The condition commonly begins in childhood, with 60% of cases appearing before the age of one and 90% manifesting by the age of five. Children with atopic dermatitis have a higher risk of developing additional health issues compared to their peers without the condition. They are more likely to experience food and environmental allergies (15% vs. 4%), asthma (25% vs. 12%), and allergic rhinitis (34% vs. 14%). Furthermore, atopic dermatitis is associated with an increased likelihood of ear infections (27% vs. 22%), streptococcal pharyngitis (8% vs. 3%), and urinary tract infections (8% vs. 3%) [5,6].

From a pathophysiological perspective, the onset of atopic dermatitis is a complex process. It is widely accepted that genetic, immunological, and environmental factors are the primary determinants of the disease [7]. Numerous genes have been associated with the pathogenesis of AD, which has led to the hypothesis that genetic predisposition plays a pivotal role in the disease’s manifestation and lesion development. The genes primarily implicated are either those that encode proteins instrumental in maintaining the skin barrier or those involved in immune regulation [8]. The filaggrin (*FLG*) gene is strongly associated with the development of AD, given its pivotal role in maintaining the skin barrier. Mutations in this gene may lead to increased permeability to allergens and irritants, as well as an increased trans-epidermal water loss [8]. Similarly, the Kallikrein-Related Peptidase 7 (KLK7) gene encodes a protease involved in the desquamation process. The overexpression or increased activity of this gene can cause skin barrier dysfunction and inflammation, which may lead to the development of both acute and chronic phases of AD [9]. Moreover, the disruption of the stratum corneum allows for the penetration of allergens, microbes, and irritants, which, in turn, trigger and perpetuate immune responses [10]. During the acute phase of AD, there is a predominance of Th2, Th22, and Th17 cytokines, as well as inflammatory cytokines such as IL-4, IL-5, IL-13, IL-17, and IL-22. In chronic AD, a shift towards a Th1 (T-helper cell type 1) predominant response contributes to the establishment of chronicity and persistence of the disease [11]. It is evident that stress, in conjunction with a multitude of environmental stimuli, has the potential to influence and exacerbate the severity of symptoms, thereby precipitating exacerbations. Irritants, such as detergents, perfumes, and specific fabrics (e.g., wool), and weather conditions, including low humidity and extreme temperatures, have the potential to exacerbate the condition of the skin and triggering flare-ups. It is well documented that common allergens such as dust mites, pollen, pet dander, and molds can elicit an immune response, thereby exacerbating the inflammatory process [12,13].

The clinical presentation of atopic dermatitis is highly variable, with skin lesions displaying a wide array of different morphological features. The most common manifestations of atopic dermatitis include erythema, edema, papules, vesicles, lichenification, dryness, and scaling, accompanied by intense pruritus. The condition has a significant impact on the patient’s quality of life, with the persistent and often severe itchiness, often leading to the “itch-scratch” cycle, which has the potential to exacerbate the condition and further disrupt the epithelial barrier. The perpetuation of the “itch-scratch” cycle may result in the development of lichenification and secondary infection of the lesions due to the compromised skin barrier. This is particularly susceptible to opportunistic pathogens such as *S. aureus.* The clinical features of atopic dermatitis may also differ in relation to the age of the patient. Indeed, in infants, lesions tend to appear predominantly on the face, scalp, and extensor surfaces of the limbs. In contrast, in older children and adults, the flexural areas, including the inner elbows, behind the knees, and around the neck, are the areas most commonly affected [14]. Moreover, atopic dermatitis is typified by two distinct phases: an acute phase and a quiescent phase. The acute phase is characterized by periods of exacerbation, whereas the quiescent phase is defined by periods of remission with a relative reduction in symptomatology. During this phase, the symptoms of dryness and scaling of the skin typically persist, even when the other symptoms have abated. The prolonged inflammation and healing processes may result in the manifestation of lichenification of the skin and hyper/hypo-pigmentation as sequelae. Eczematous plaques, which are well-demarcated and hyperkeratotic, can manifest in flexural and interdigital areas [14].

The management of atopic dermatitis necessitates a comprehensive, step-by-step approach that is tailored to the severity of the condition. For all individuals, the cornerstone of care and prevention of flare-ups is daily bathing or showering, immediately followed by the application of moisturizers or emollients. Furthermore, it is imperative to refrain from exposure to potential triggers, including irritants, aeroallergens, food allergens, and extreme temperatures or humidity levels. In cases of mild atopic dermatitis, treatment typically involves the intermittent use of low- to medium-strength topical corticosteroids (TCS) as required. In cases of moderate to severe atopic dermatitis, the regular use of medium-strength topical corticosteroids is recommended. Furthermore, the United States Food and Drug Administration (FDA) has approved the use of topical calcineurin inhibitors (TCIs), including pimecrolimus and tacrolimus, as well as crisaborole, as alternative treatments. Topical anti-inflammatory therapies may be applied either preventively (once or twice daily or weekly, depending on the medication’s strength) or during a flare-up. In cases of severe or treatment-resistant AD, it is recommended that patients seek the advice of a dermatologist or allergist. In cases where local treatments are ineffective or unsuitable, systemic treatments, such as dupilumab or other immunosuppressive therapies, may be required as chronic or repeated use of systemic corticosteroids may lead to various side effects, these medications may be employed on a temporary basis as a transition to more sustainable long-term therapies, thus reducing the risk of adverse events [7]. As a result, numerous researchers have focused on advancing innovative pharmaceutical technologies to address the need for frequent drug administration [15]. Additional options for the management of moderate-to-severe AD include phototherapy and the use of systemic medications such as cyclosporine, methotrexate, azathioprine, or mycophenolate mofetil [16].

## 3. Atopic Keratoconjunctivitis

Atopic eye disease represents a complex group of ocular pathologies that frequently occurs in patients with atopic dermatitis. The etiology of atopic eye disease in patients with AD can be attributed to three primary causes (Table 1) [17]. The first is ocular allergies in individuals with a genetic predisposition to allergic reactions (as atopic dermatitis is itself an allergic disease). The second is adverse reactions to medications used to treat atopic dermatitis (such as dry eye and dupilumab-associated dry follicular conjunctivitis). The third is the localization of atopic dermatitis to the ocular surface itself, known as atopic keratoconjunctivitis.

Atopic keratoconjunctivitis (AKC) is a chronic inflammatory condition affecting both the conjunctiva and eyelids, frequently associated with atopic dermatitis. While the majority of cases of atopic dermatitis are identified by the age of five, AKC typically emerges between the second and fifth decades of life. The primary symptom is intense itching, which may be seasonal or year round, and is accompanied by tearing, mucus discharge, redness, blurred vision, light sensitivity, and pain [18].

The clinical signs include alterations to the skin around the eyes, such as redness, flaking, and scaling dermatitis. Complications may also arise, such as cicatricial ectropion and lagophthalmos, due to eyelid thickening and hardening. Additional findings may include the presence of lateral canthal ulcers, fissures, madarosis, a reduction in eyelash density, and the onset of meibomian gland inflammation. The conjunctiva may display papillary reactions, follicles, or pale swelling, with papillary hypertrophy being more pronounced in the lower fornix in comparison to vernal keratoconjunctivitis (VKC). Additionally, subepithelial fibrosis, fornix shortening, and symblepharon may occur. The primary causes of vision loss in this context are corneal issues, including punctate epithelial keratopathy, epithelial defects, scarring, microbial ulceration, and neovascularization. Cataracts, which are frequently anterior or subcapsular, typically exhibit a Maltese cross pattern [19].

AKC is characterized by both type I and IV hypersensitivity, with the presence of mast cells and eosinophils in the conjunctival epithelium. This results in reduced tear stability, decreased corneal sensitivity, and elevated inflammatory biomarkers. An increase in mononuclear cells, fibroblasts, and collagen is observed in both the conjunctiva and substantia propria, accompanied by elevated levels of inflammatory and immune mediators. Analyses of the blood and tears reveal elevated levels of immunoglobulin E (IgE), eosinophil cationic protein, and other inflammatory markers [18].

The diagnosis is clinical, highlighting all the above changes; however, in vivo confocal microscopy, a tool that allows real-time visualization of the corneal and conjunctival layers, is extremely helpful in diagnosing and monitoring the condition. It allows the detection of subclinical changes in the corneal and conjunctival epithelium, including inflammatory cell infiltration, tissue damage, and changes around the conjunctival papillae, corneal epithelium, sub-basal nerve structure, and goblet cell [20].

The treatment plan typically involves a combination of environmental adjustments and pharmacological agents. It is of the utmost importance to avoid irritants, and the use of skin testing may prove beneficial in identifying potential triggers. Topical vasoconstrictors and antihistamines provide temporary symptomatic relief; however, they do not address the underlying immune mechanisms. The use of antihistamines and short-term topical steroids, employed with caution, has been demonstrated to be an effective approach for the management of symptoms. Mast cell stabilizers, such as cromolyn and lodoxamide, have been demonstrated to be beneficial for long-term control. Topical ciclosporin A or tacrolimus has also been shown to reduce steroid dependency. In cases of sight-threatening complication, oral steroids may be required. The use of artificial tears can prevent epithelial defects in persistent corneal staining, while antiviral agents are effective in managing HSV infections. It is recommended that steroid-sparing strategies are adopted at the earliest opportunity in order to avoid long-term complications [18]. Evaluating the pattern of allergen sensitization has the potential to guide future recommendations for avoidance strategies and immunotherapy. In this context, it represents a possible approach that warrants further investigation, as it could eventually contribute to reducing corticosteroid use, thereby potentially mitigating disease severity and associated visual complications [21]. Moreover, to prevent the risk of iatrogenic damage to the ocular surface, particularly in cases of prolonged topical therapy, it may be advisable to opt for preservative-free formulations, carefully weighing the risks and benefits [22].

## 4. Human Microbiota and Probiotics

The term “microbiota” is typically used to refer to the collection of diverse microorganisms that reside within the human body or on its surface, including the gut, oral cavity, skin, and eyes. This intricate ecosystem encompasses a multitude of microorganisms, including commensal, symbiotic, and even pathogenic species, numbering in the trillions [3]. It is estimated that a healthy human gut hosts at least 800 different bacterial species, with an average of at least 10^9^ bacterial cells per gram of stool [3]. Factors such as external environment, diet, and lifestyle have been identified as playing a pivotal role in influencing the composition and health of the microbiome. Recent research has underscored the pivotal role of the microbiome in influencing health and contributing to disease. The dynamic nature of these microorganisms, encompassing their quantity, composition, and activity, has been demonstrated to affect the onset and progression of various conditions, including cancer, metabolic and cardiovascular disorders, and psychological illnesses such as schizophrenia. Moreover, imbalances in the microbial community, or dysbiosis, have been linked to inflammatory bowel disease, multiple sclerosis, diabetes, allergies, asthma, autism, and cancer [2]. This association has prompted interest in the potential of probiotics as a means of positively influencing the microbiota for the prevention and treatment of diseases [22].

In 2001, the Food and Agriculture Organization of the United Nations (FAO) and the World Health Organization (WHO) defined probiotics as “live microorganisms which, when administered in adequate amounts, confer a health benefit on the host” [23]. They can be administered in the form of drugs or foodstuffs [24]. An increasing body of evidence indicates that probiotics are being incorporated into functional foods, dairy products, and other dietary supplements with the objective of maintaining and promoting human health [25]. The efficacy of probiotics is contingent upon their ability to survive in the novel host environment, which encompass their capacity to withstand the harsh conditions of the gastrointestinal tract, including exposure to gastric acid, biliary salts, and digestive enzymes. It is of paramount importance to ensure that a viable number of living probiotics reach their target site in the lower GI tract in order to achieve the desired probiotics formulation and efficacy. Consequently, the different delivery routes play a crucial role in the usage of probiotics as potential therapies [26].

Probiotic products have historically been administered in the form of capsules, tablets, or powders. While these delivery routes provide a convenient method of consumption, they do not always protect the probiotics from the acidic environment of the stomach and the action of bile salts. A notable advancement in the mode of delivery of probiotic formulations has been the emergence of microencapsulation technology. This technology provides beneficial bacteria with a protective coating, shielding them from the harsh environment of the gastrointestinal tract. This has been shown to significantly enhance their viability, particularly when combined with other prebiotic products [27,28]. These innovative delivery methods (synbiotics), which combine probiotics with prebiotics to enhance bacterial survival and promote colonization, are rapidly gaining popularity, along with food-based delivery systems where probiotics are incorporated into everyday food items [29]. Although it may appear to be a relatively straightforward process, the evaluation of probiotics survival curves and related health benefits necessitates the implementation of rigorous in vivo and in vitro testing procedures. This is in order to ensure that a sufficient number of viable cells reach their target zone, where they can effectively perform their desired function [23].

The majority of commercially available probiotics (Table 2) are derived from bacterial strains belonging to the genera *Lactobacillus* and *Bifidobacterium* [30,31,32]. Among the lactic acid bacteria (LAB), genera such as *Lactobacillus*, *Lactococcus*, *Enterococcus*, and *Pediococcus* are of particular significance due to their contributions to the gut microbiome, antimicrobial activity, and immunomodulatory properties. These characteristics position them as a promising and safe probiotic when administered in appropriate doses [33].

One of the primary mechanisms through which probiotics exert their beneficial effects on the host is their microbiological functionality. Bifidobacteria and lactic acid-producing bacteria may, in fact, compete with other pathogenic microorganisms for nutrients and adhesion sites, improve epithelial gut barrier function, degrade toxins receptors, and regulate the overall gut microbiota composition [40]. A recent study demonstrated that a probiotic formulation containing Lactobacilli and Bifidobacteria species could also enhance the expression and assembly of tight junction proteins such as occludin, claudin, and zonula occludens (ZO-1). This reinforcement of the tight junctions helps to maintain the integrity of the intestinal barrier and to reduce permeability to toxins and pro-inflammatory mediators [41]. Moreover, it has been demonstrated that certain strains of LAB can facilitate the turnover and repair of epithelial cells in the gut lining. This is achieved by inducing ERK and FAK phosphorylation, which subsequently triggers the proliferation and migration of intestinal epithelial cells in murine models [42]. This may assist in maintaining a robust barrier function and facilitating rapid recovery from damage caused by infections or inflammatory insults.

Probiotics also play a role in the fermentation of undigested carbohydrates, producing short-chain fatty acids (SCFAs) such as butyrate, propionate, and acetate, which do not exceed the length of C6 [43]. It is well documented that butyrate serves as the primary energy source for colonic epithelial cells, accounting for approximately three-quarters of their energy input. Additionally, it has been demonstrated that probiotics can enhance the expression of tight junction proteins, thereby strengthening the intestinal barrier and reducing exposure to pro-inflammatory molecules and subsequent gut inflammation [44,45].

From an immunological standpoint, probiotics interact with the gut-associated lymphoid tissue (GALT) to modulate the immune response by stimulating B lymphocytes to produce immunoglobulin A (IgA), an antibody class that is vital for immune function as it prevents pathogens from adhering to the gut lining. Chronic inflammation has the potential to damage the intestinal barrier; however, probiotics can help to mitigate this damage by inhibiting the production of pro-inflammatory cytokines, such as TNF-α and IL-6, and by producing anti-inflammatory molecules, such as IL-10 [46]. Furthermore, a novel probiotic formulation comprising *Bacillus subtilis* DE111 has demonstrated encouraging outcomes as an immunomodulator, markedly reducing the levels of CD3+ T cells, CD25+FoxP3+ T regulatory cells and CD8+ cytotoxic T cells. This may assist in maintaining immune tolerance and preventing excessive inflammatory responses that could potentially harm the intestinal barrier [47].

Despite the extensive research on bacterial probiotics, the potential of yeasts as probiotics remains relatively underexplored. Nevertheless, emerging evidence indicates that specific yeast strains may confer health benefits. Of these, *Saccharomyces cerevisiae* and *Saccharomyces boulardii* have been the subject of the most extensive study with regard to their probiotic attributes. The beneficial effects of *S. boulardii* are largely attributed to its capacity to modulate host immune responses and competitively inhibit pathogenic bacteria [37,48,49]. Furthermore, recent research has identified additional yeast species with potential probiotic properties. For example, *Kluyveromyces marxianus*, a yeast commonly associated with kefir grains, has been demonstrated to play a pivotal role in the development of desirable flavor profiles in milk kefir [50]. Similarly, *Pichia kudriavzevii*, which is frequently found in the environment and in naturally fermented foods, such as fruits, cocoa, coffee beans, cheese, and yoghurt, has demonstrated potential as a biocontrol agent by suppressing the growth of various plant pathogens [51]. In our recent study, we isolated and characterized a yeast strain (*Pichia kudriavzevii*), along with two lactic acid bacteria (*Lactococcus lactis* subsp. *hordniae* and *Lactococcus lactis* subsp. *lactis*), from traditional homemade kefir. The probiotic potential of these strains was evaluated through in vitro and in vivo studies. The findings revealed that the isolates exhibited promising probiotic properties and demonstrated favorable safety profiles, thereby supporting their suitability as starter cultures for the production of probiotic food products [52].

## 5. The Role of Microbiota in Atopic Dermatitis and Atopic Ocular Diseases

In recent years, there has been a growing body of evidence that highlights the pivotal role of microbiota in the development and exacerbation of atopic dermatitis. Particular attention has been paid to the contribution of microbial imbalance or dysbiosis to this process [53].

There are notable differences in the gut microbiota of patients with atopic dermatitis when compared to that of a healthy individual. These alterations encompass both changes in the relative abundance of individual microbial species (with some populations being present in different proportions compared to healthy individuals) and more general alterations in the diversity of microbial species composing the microbiota (AD patients exhibit lower diversity) [54].

Among bacterial families with an altered abundance in the gut microbiome, those belonging to the Actinobacteria phylum (such as *Propionibacteriaceae*, *Actinomycetaceae*, *Coriobacteriaceae*, and *Bifidobacteriaceae*) are typically reduced in individuals with atopic dermatitis. Conversely, the relative abundance of *Bacteroidaceae* (Bacteroidetes) and *Alcaligenaceae* (Proteobacteria) tends to increase. Furthermore, specific bacterial genera (e.g., *Coprococcus*, *Bifidobacterium*, and *Propionibacterium*) that are known for their anti-inflammatory properties or roles in immune homeostasis, and which could potentially confer protection against AD, are either absent or present in reduced proportions [54]. A recent systematic review examined the bacterial colonization patterns in AD, with a particular focus on Bifidobacteria, Clostridia, and Lactobacilli. The distribution of Bifidobacteria subspecies demonstrated disparate patterns, with some exhibiting an augmented presence and others a reduction in children with AD. Some studies have indicated that elevated levels of Clostridia are associated with an increased risk of AD, whereas other studies have not identified a significant correlation. The colonization of the gut by *Lactobacillus paracasei* has been observed to lower the risk of developing AD in certain studies. However, other studies have reported either no effect or a reduction in the colonization of the gut by *Lactobacillus* in patients with AD [55,56].

Regarding microbial diversity, a reduction has been observed in infants with atopic eczema, particularly within the Bacteroidetes phylum and Bacteroides genus at one month, and within the Proteobacteria phylum at twelve months. This suggests that a reduction in microbial diversity during the first months of life may be linked to subsequent atopic eczema development, rather than to any specific species [51,57]. Furthermore, a comparison of infants with atopic dermatitis to controls revealed lower bacterial cell counts in their feces, accompanied by a notable reduction in the expression of genes involved in oxidative phosphorylation and antigen processing. This suggests a potential association between bacterial genes affecting immune function and the development of AD [58].

The pattern of gastrointestinal microbiota colonization appears to be influenced by the mode of delivery at birth. The colonization rates of beneficial bacteria, including *Bifidobacteria*, *Bacteroides*, and *Escherichia coli*, are observed to be lower in infants delivered via cesarean section compared to those delivered vaginally, with the latter group exhibiting higher colonization rates of Clostridia. The differences in gut microbiome development contribute to alterations in the intestinal environment. Infants with atopic dermatitis display mature short-chain fatty acid (SCFA) profiles prior to 12 months of age but experience delayed maturation thereafter. The pathways involved in butyrate production, including those mediated by glutamate decarboxylase and succinate dehydrogenase, are typically less active in populations with atopic dermatitis. *Bacteroides fragilis*, a key bacterium, has been linked to deficient butyrate production and elevated acetate levels in infants with atopic dermatitis. Furthermore, bacterial genes encoding G-protein coupled receptor 109A (Gpr109a) and peroxisome proliferator-activated receptor-γ (Pparg) are downregulated in AD, which correlates with reduced butyrate levels and impaired gut health [59].

The gene expression profile of the AD microbiota also shifted towards metabolic activity focused on oxidative stress responses, as opposed to the typical biosynthetic pathways for amino acids, nucleotides, peptidoglycans, and cofactors. This shift is likely the result of a dysregulated gut environment, characterized by heightened inflammation, and an increased availability of host-derived nutrients. Such nutrient availability may suppress fundamental biosynthetic gene activity across the microbiome, while selective pressures, such as the presence of specific molecules like N-acetylgalactosamine (GalNAc), favor species capable of metabolizing them. Such environmental alterations can intensify the degradation of gut tissue, thereby precipitating further dysbiosis and microbial dysfunction [60].

**Figure 1:**
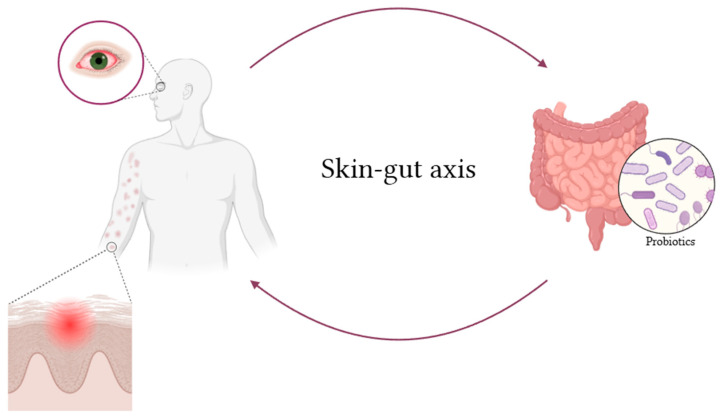
This schematic image illustrates the two-way interaction between the gut microbiota and the skin. In the case of gut dysbiosis and altered microbiota, inflammatory skin conditions can occur, which probiotic supplementation can positively modulate by reducing inflammation. The figure was created with Biorender.com.

The concept of a “skin-gut axis” (Figure 1 and Table 3) is gaining traction in the scientific community, whereby alterations in the gut microbiota can exert an influence on cutaneous health. An imbalance in the gut microbiome, frequently characterized by a reduction in beneficial bacteria such as Bifidobacteria and Lactobacilli, may result in systemic inflammation that impacts the skin [61]. The skin microbiome plays a pivotal role in maintaining the integrity of the skin barrier and modulating the immune response. In individuals with atopic dermatitis, the integrity of the skin barrier is frequently compromised, increasing susceptibility to infections and allergens that can trigger or exacerbate disease flares [62]. A significant body of research has demonstrated that individuals with atopic dermatitis exhibit a markedly altered skin microbiome in comparison to healthy individuals. One of the most salient findings in AD is the overabundance of *Staphylococcus aureus*, a bacterium known to be pathogenic and capable of exacerbating inflammation. This is accompanied by a reduction in beneficial bacteria, including species such as *Diphtheroids* and *Lactobacilli*. *Staphylococcus aureus* produces exotoxins that can directly contribute to cutaneous inflammation and trigger the release of pro-inflammatory cytokines, such as interleukin (IL)-4, IL-5, and IL-13, which are characteristic of the Th2-driven immune response observed in AD [63,64].

Recent research has highlighted the important role of the gut microbiota in the development and progression of several eye diseases. The bidirectional interaction between the gut and the eye, known as the gut–eye axis, underscores this link. In a healthy state, intestinal homeostasis supports ocular health by modulating the immune system and producing anti-inflammatory compounds such as short chain fatty acids (SCFAs), bacteriocins, secondary bile acids, indoles, and polyamines. However, in cases of dysbiosis, the proliferation of pro-inflammatory bacteria disrupts the intestinal barrier, leading to metabolic endotoxemia, systemic inflammation, and impaired ocular health [4,65]. Furthermore, the ocular surface microbiota plays a pivotal role in maintaining local homeostasis and safeguarding against ocular diseases. Alterations in the typical microbial composition of the eye have been associated with an array of conditions, including blepharitis, conjunctivitis, keratitis, trachoma, and dry eye syndrome (DED), as evidenced by a multitude of studies [4]. Inada et al. investigated the ocular surface microbiota in patients with refractory allergic conjunctival diseases (ACDs), including VKC and AKC, who were being treated with topical tacrolimus. A significant difference was observed in the β-diversity of the ocular surface microbiota between these patients and the control group [66]. Also, the research team led by Hur investigated ocular microbiota in patients with atopic keratoconjunctivitis and compared it to that of healthy individuals. The results demonstrated significant discrepancies in microbiome diversity and composition between the two groups. In particular, patients with AKC exhibited greater variation in conjunctival taxonomic composition, with notable differences in the relative abundance of genera, including *Ralstonia*, *Staphylococcus*, *Pseudomonas*, *Proteus*, *Haemophilus*, and *Bifidobacterium* (*p* < 0.05). Furthermore, the β-diversity was found to be significantly higher in the AKC group in comparison to the healthy control group [67].

**Table 3:** Gut–skin–eye axis.

Gut Microbiota	Effect on Skin and Eye	Reference
Production of metabolites	Microbial metabolites (e.g., short-chain fatty acids [SCFAs]) produced by gut bacteria, regulate immune tolerance, and inflammation, and impact on skin conditions and eye inflammation.	[68]
Immune system modulation	The immune system acts as the mediator between the gut, skin, and eyes by circulating cytokines, metabolites, and immune cells.	[68]
Systemic inflammation regulation	Intestinal permeability (“leaky gut”) allows inflammatory molecules to enter the bloodstream, influencing systemic inflammation, which, in turn, can exacerbate skin and eye pathologies.	[68]

## 6. The Role of Probiotics in Atopic Dermatitis and Atopic Ocular Diseases

In recent years, a substantial corpus of the literature has been published on the potential therapeutic role of probiotics in the management of AD. The role of probiotics in atopic ocular disease represents an emerging area of research that has gained interest due to the potential of probiotics to influence the immune system and microbiome, which may play a critical role in the pathogenesis of various allergic conditions, including ocular diseases associated with atopy [69]. The pathways of probiotic-mediated immune responses are resumed in Table 4 [23,70].

Currently, a significant number of probiotic strains and formulations have been investigated with a view to establish their potential therapeutic value in the management of AD. *Lactobacillus plantarum* and *Lactobacillus fermentum*, administered at various dosages, are currently among the most popular probiotics [71]. Furthermore, *Enterococci*, *Bifidobacteria*, and other *Lactobacillus* strains are commonly utilized as probiotics in the management of atopic dermatitis [69]. However, it is currently unclear which strains and dosages are the most efficacious across diverse age and ethnic cohorts.

Some studies have concentrated on specific *Lactobacillus* strains and their physiological and pathological role in the mitigation of AD symptoms. Specifically, species such as *L. reuteri* and *L. plantarum CJLP133* have been observed to downregulate the expression of *TARC*, a pivotal chemokine implicated in the development of atopic dermatitis [72,73]. Similarly, *Lactococcus lactis* LB 1022 has been demonstrated to possess anti-allergic and anti-inflammatory properties, in addition to the capacity to modulate symptoms characteristic of atopic dermatitis [74].

Furthermore, the potential role of Bifidobacteria in the management of AD has also been the subject of considerable interest. Akay et al. observed a reduced prevalence of *Bifidobacterium longum* in children with AD, which suggests that this particular strain may have a protective role [38]. Similarly, a clinical trial documented that the administration of the *Bifidobacterium breve* M-16V strain resulted in an increase in *Bifidobacterium* abundance in the fecal microbiota, which, in turn, alleviated allergic symptoms in infants with AD. However, it would appear that not all strains of Bifidobacteria are equally beneficial for patients with atopic dermatitis [39]. As reported by Gore et al. (2008), an association exists between atopic dermatitis and the presence of *Bifidobacterium pseudocatenulatum* in feces, indicating a potential exacerbating effect on the symptomatology [40]. In contrast, another study found that the intake of probiotics, specifically *Lactobacillus acidophilus* NCFM and *Bifidobacterium animalis subsp. lactis* Bi-07, did not result in a statistically significant alteration in fecal microbiota composition in children with AD [41]. These examples illustrate the complex relationship between Bifidobacterium species and AD, indicating the potential for diverse roles among different strains.

A systematic review and meta-analysis by Zuccotti et al. evaluated the effect of probiotic supplementation during pregnancy and early infancy on the prevention of atopic diseases. With regard to atopic dermatitis, data from 4755 children (2381 in the probiotic group and 2374 in the control group) were analyzed. In most of the trials, probiotic supplementation started during pregnancy, except in the Prescott et al. and Taylor et al. trials, where the infants of atopic mothers were given probiotics within 48 h of birth. A lower percentage of children in the probiotic group developed eczema than in the control group (28.22% vs. 35.67%). The number needed to treat (NNT) was 13, meaning that 13 children needed probiotic supplementation to prevent one case of eczema. The probiotic strains used varied. Four trials tested mixtures of Lactobacilli and Bifidobacteria given to pregnant women and infants, three trials tested single strains of each bacterium, and ten trials focused on different strains of Lactobacilli. Sub-meta-analyses showed that probiotic mixtures significantly reduced the risk of eczema. However, no significant effect was seen in trials that used only Lactobacilli or Bifidobacteria. Age-specific analyses showed that probiotics were effective in preventing eczema in children up to 24 months of age, with a reduced effect after this age [75].

Although research on the use of probiotics for atopic ocular disease is still in its infancy, a number of studies and clinical trials have indicated the potential benefits of this approach. Iovieno et al. evaluated the efficacy of *Lactobacillus acidophilus* eye drops in the management of symptoms associated with vernal keratoconjunctivitis. The study cohort comprised seven patients (mean age 11.8 ± 4.3 years; five males, two females) with mild to moderate VKC. The study participants were administered *Lactobacillus acidophilus* (2 × 10⁸ CFU/mL) diluted in saline as eye drops four times daily for four weeks, with the drops instilled in both eyes. Among the six patients who completed the study, a significant reduction in symptoms was observed after both two weeks [76].

Dennis-Wall et al. conducted a double-blind, placebo-controlled, randomized clinical trial to investigate the impact of a probiotic blend (*Lactobacillus gasseri* KS-13, *Bifidobacterium bifidum* G9-1, and *Bifidobacterium longum* MM-2) on quality of life in individuals suffering from seasonal allergies. The trial involved 173 participants (mean age: 27 ± 1 years) who reported experiencing seasonal allergies and were randomly assigned to either the probiotic group or a placebo group for an eight-week intervention during the spring allergy season. The primary outcome was evaluated using the Mini Rhinoconjunctivitis Quality of Life Questionnaire (MRQLQ), with weekly score collection throughout the study period. Secondary outcomes were directed towards immunological parameters, including serum IgE levels and the percentage of regulatory T cells (Tregs), which were measured in a subset of participants at baseline and during the pollen season’s peak (week 6). The probiotic group exhibited a notable enhancement in MRQLQ scores from the baseline to the pollen peak, in comparison to the placebo group (mean difference: −0.68 ± 0.13 vs. −0.19 ± 0.14; *p* = 0.0092). This observation suggests an augmented rhinoconjunctivitis-specific quality of life. Although both groups exhibited augmented serum IgE and Treg percentages over time, these alterations were not statistically distinct between the probiotic and placebo groups. The findings suggest that this probiotic combination can effectively enhance the quality of life for individuals with seasonal allergies. However, the precise biological mechanism remains unclear [77].

A study by Nocerino et al. examined the effects of various formulas on the development of atopic conditions, including eczema, urticaria, asthma, and rhinoconjunctivitis, as well as the time needed to achieve immune tolerance in children with IgE-mediated cow’s milk allergy (CMA). Over a 36-month period, 365 children were randomly assigned to one of six formula groups, including an extensively hydrolyzed casein formula with *Lactobacillus rhamnosus* GG (EHCF + LGG), a rice hydrolyzed formula, a soy formula, an extensively hydrolyzed whey formula (EHWF), and an amino acid-based formula. The study revealed that the EHCF + LGG group exhibited the lowest prevalence of atopic conditions, a finding that was significantly lower than that observed in the other groups. Furthermore, the children in this group demonstrated a greater propensity to achieve immune tolerance, and at an earlier point in time, in comparison to those in the other groups. These findings suggest that EHCF + LGG may reduce the risk of atopic conditions and accelerate immune tolerance, thereby making it a potentially advantageous option for children with IgE-mediated CMA [78].

A recent study on mandarin orange yoghurt, which contains nobiletin and β-lactoglobulin, has yielded promising results. Following a two-week consumption period, participants exhibited notable reductions in symptoms of allergic conjunctivitis such as redness, swelling, itching, and temperature compared to a control group that consumed yoghurt without mandarin orange [79].

Although the potential benefits of probiotics for individuals with atopic dermatitis and atopic ocular diseases are promising, several challenges remain. There is a paucity of clinical trials that have specifically addressed the role of probiotics in atopic ocular disease. The majority of research has concentrated on systemic atopic conditions or generalized allergic responses. The effects of probiotics are highly strain-dependent. The effects of probiotics on immune modulation may vary depending on the specific strain, and not all strains may be beneficial for the treatment of allergic conditions (among lactic acid bacteria, genera such as *Lactobacillus, Lactococcus, Enterococcus*, and *Pediococcus* are generally thought to be particularly effective) [80]. The optimal dosages and forms of probiotics for the management of atopic dermatitis and atopic ocular diseases remain to be established. Probiotic dose, treatment duration and patient selection criteria are variables that can significantly influence the results of a clinical trial. As a result, the variation in these variables makes it difficult to compare results from different trials. For this reason, it is important that further research is conducted on this topic and that consistent inclusion criteria are established to facilitate comparisons between different trials. Further research is required in order to ascertain the most efficacious protocols for supplementation. In particular, further research is required to establish clear evidence on the role of probiotics in atopic ocular disease. It is imperative that well-designed clinical trials be conducted that specifically address ocular symptoms, dose–response relationships, and the specific strains of probiotics involved. Furthermore, an investigation of the gut–eye axis and the microbiome’s role in ocular inflammation may provide new insights into the integration of probiotics into the treatment of allergic ocular diseases.

## 7. Conclusions

Human microbiota plays a pivotal role in maintaining health and preventing disease. In particular, the microbiota plays a pivotal role in the pathogenesis of atopic dermatitis and atopic ocular diseases, exerting influence over immune responses, skin barrier function, and inflammation. A microbial imbalance, or dysbiosis, in the skin and gut microbiomes has been identified as a potential factor in the development and exacerbation of these conditions. Probiotics, which modulate microbiota and enhance immune responses, represent a promising tool for improving health and treating various conditions, particularly those related to the gut, skin, and immune system, including both cutaneous and ocular manifestations in atopic disease. Despite the considerable advances that have been made in our understanding of probiotics and their impact on the human body, it is clear that further research is required if we are to fully comprehend their potential therapeutic applications and to optimize their use in clinical settings. Nevertheless, further research is required to elucidate the precise mechanisms involved and to ascertain the most efficacious strategies for microbiota modulation in these conditions. As microbiome science continues to advance, probiotics may eventually become a fundamental component of personalized medicine and preventive health care.

## Figures and Tables

**Table 1:** Classification of atopic eye disease in patients with atopic dermatitis.

Category	Condition
Ocular allergies	Seasonal allergic conjunctivitis Perennial allergic conjunctivitis Vernal keratoconjunctivitis
Adverse reactions to medication	Dry eye Dupilumab-associated conjunctivitis
Ocular localization of atopic dermatitis	Atopic keratoconjunctivitis

**Table 2:** Common probiotic species and their beneficial effects.

Probiotic Species	Diseases	Health Benefits	References
*Bifidobacterium bifidum*	Intolerance to lactose; diarrheal infections	Use lactose; inhibitions of *E. coli*, *Salmonella* and *C. difficile*	[34]
*Lactobacillus rhamnosus GG*	Food allergies, cancer, diarrheal infections	Aid in reducing intestinal inflammation and hypersensitivity reactions in infants who have food allergies; reduces pancreatic cancer risk; inhibitions of *E. coli*, *Salmonella* and *C. difficile*	[35]
*Lactobacillus acidophilus*	Enteric infections	Inhibition of *S. aureus* growth and has a strong anti-inflammatory effect	[36]
*Saccharomyces boulardii*	Gut infections, immune enhancement	Prevents digestive tract infections (e.g., *C. difficile*) and regulates immune response	[37]
*Saccharomyces cerevisiae*	Glycemic imbalance, immune enhancement	Promotes optimal metabolic health (diabetes management), upholds immune function, and lowers oxidative stress.	[38]
*Pichia kudriavzevii*	High cholesterol levels	Improves digestive health and reduces cholesterol levels	[39]

**Table 4:** Pathways of probiotic-mediated immune responses.

Pathway	Mechanism	Immune Response/Effect on Skin and Eye
Modulation of gut microbiota	Probiotics restore microbial balance by promoting beneficial bacteria and suppressing pathogens.	Improves gut barrier integrity, reduces inflammation, and supports a healthy immune system.
Production of metabolites	Probiotics produce metabolites (e.g., SCFAs, lactic acid) that modulate immune cell functions.	Enhances anti-inflammatory responses and promotes regulatory T cell (Treg) development.
Interaction with gut epithelium	Probiotics strengthen the intestinal epithelial barrier by increasing tight junction protein expression.	Reduces gut permeability and prevents translocation of harmful antigens or pathogens into the bloodstream.
Direct interaction with immune cells	Probiotics interact with dendritic cells, macrophages, and T cells via Toll-like receptors (TLRs).	Promotes the maturation of dendritic cells, enhances phagocytosis, and induces Treg cells to regulate immunity.
Secretion of immunomodulatory molecules	Probiotics secrete compounds like bacteriocins, exopolysaccharides, and enzymes.	Modulates the production of cytokines (e.g., IL-10, IL-6, and TNF-α), balancing pro- and anti-inflammatory responses.
Activation of gut-associated lymphoid tissue (GALT)	Probiotics stimulate Peyer’s patches and lymphoid follicles in the gut.	Enhances the production of IgA, which supports mucosal immunity and protects against pathogens.
